# A Hidden Neighbor: A Rare Benign Paratesticular Mass Presenting as a Testicular Tumor

**DOI:** 10.7759/cureus.114623

**Published:** 2026-08-16

**Authors:** Girish Bakhshi, Aayushi A Agre, Yashika Gupta, Shivangi Tiwari, Ranjitha R Maiyya

**Affiliations:** 1 General Surgery, Grant Government Medical College, Mumbai, IND

**Keywords:** benign, leiomyoma, non specific clinical presentation, paratesticular mass, testis sparing surgery

## Abstract

Paratesticular masses are uncommon lesions arising from structures adjacent to the testis, including the epididymis, spermatic cord, and tunica vaginalis, and encompass a wide spectrum of benign and malignant pathologies. Due to their rarity and nonspecific clinical presentation, accurate preoperative diagnosis can be challenging. Ultrasonography serves as the primary imaging modality for initial evaluation, while cross-sectional imaging may further aid in lesion characterization and staging. We report a case of a 52-year-old male patient presenting with a painless scrotal swelling, in whom imaging revealed a well-defined extra-testicular mass with features suggestive of benign disease. Surgical excision was performed, and histopathological examination established the definitive diagnosis. This case underscores the importance of thorough imaging assessment in differentiating para-testicular from intratesticular lesions and guiding appropriate management. Early recognition is essential to avoid overtreatment of benign conditions and ensure timely intervention in malignant cases.

## Introduction

Paratesticular masses are uncommon, extratesticular lesions arising from the epididymis, spermatic cord, tunica vaginalis, and adjacent soft tissues, accounting for approximately 5-10% of all intrascrotal tumors [1]. These lesions encompass a wide pathological spectrum, of which nearly 70-80% are benign [2]. The most frequently reported benign paratesticular tumors include adenomatoid tumors, lipomas, leiomyomas, and inflammatory pseudotumors, while malignant lesions such as sarcomas constitute a minority of cases [2,3].

Benign paratesticular masses may originate from mesothelial, adipose, smooth muscle, or inflammatory processes [2,4]. Clinically, patients typically present with a painless, slowly enlarging scrotal swelling, and physical examination alone is often insufficient to reliably differentiate extratesticular from intratesticular pathology [5]. This diagnostic uncertainty frequently raises concern for testicular malignancy, leading to aggressive surgical management.

Given their rarity and overlapping clinical and imaging features with testicular malignancies, benign paratesticular masses are frequently misdiagnosed, potentially resulting in unwarranted radical surgery. Case reports therefore play an important role in increasing clinical awareness and reinforcing conservative, organ-preserving management strategies when appropriate [1,6].

## Case presentation

A 52-year-old male presented with a complaint of painless swelling of the left hemiscrotum, first noticed four months prior to presentation. The swelling was gradually progressive in nature. There was no history of trauma, fever, lower urinary tract symptoms, or infertility.

On physical examination, the left hemiscrotum was mildly enlarged. A well-defined, firm, non-tender mass measuring approximately 3 × 3 cm was palpated adjacent to the left testis. The overlying scrotal skin appeared normal. The right testis and hemiscrotum were unremarkable.

Routine hematological investigations, including complete blood count, were within normal limits. Semen analysis showed normal parameters. Serum tumor markers (lactate dehydrogenase (LDH), alpha-fetoprotein (AFP), and beta-human chorionic gonadotropin (β-HCG)) were all within normal reference ranges.

Ultrasonography of the bilateral inguinoscrotal region demonstrated a well-defined, round to oval, homogeneous, mildly hyperechoic extratesticular mass measuring 3 × 3 cm in the left hemiscrotum. The lesion exhibited mild internal vascularity on color Doppler imaging. There was no evidence of testicular involvement, scrotal wall invasion, or associated hydrocele.

**Figure 1:**
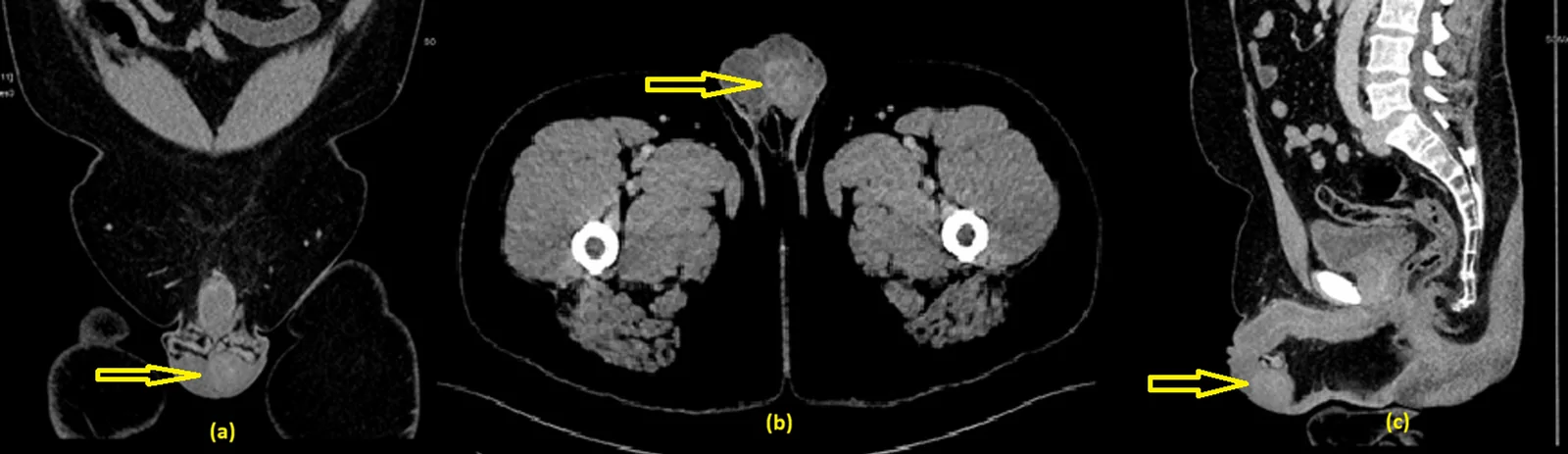
Contrast-enhanced computed tomography coronal (a), axial (b) and sagittal (c) reconstructions of abdomen and pelvis reveals a left-sided, oval, well-circumscribed, mildly enhancing extra-testicular mass (yellow arrow).

Contrast-enhanced computed tomography (CT) of the abdomen and pelvis revealed a well-circumscribed, mildly enhancing left intrascrotal mass, distinctly separate from the left testis, with no signs of local extension or distant metastasis (Figure 1).

The patient was planned for excision of the mass with a standby option of orchidectomy, if intraoperative findings suggested testicular involvement.

Following administration of spinal and epidural anesthesia, the patient was placed in the supine position. A left hemiscrotal incision was made approximately 2 cm lateral to the midline raphe and deepened up to the tunica vaginalis. A small tunica vaginalis sac containing the testis and an adjacent mass was identified. Upon opening the sac, approximately 10-15 mL of straw-colored fluid was aspirated and sent for analysis.

**Figure 2:**
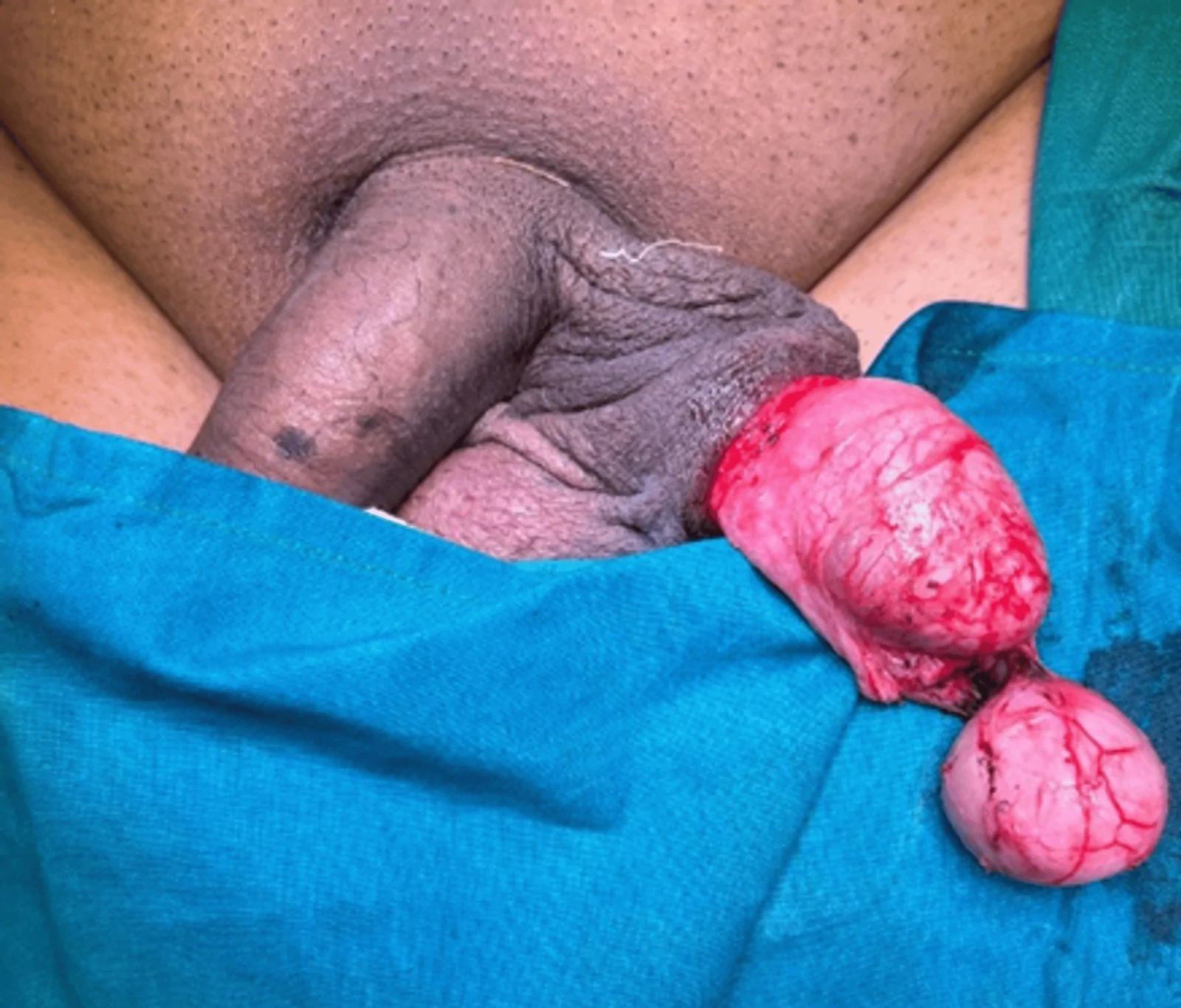
Intraoperative picture showing left-sided well-circumscribed paratesticular mass attatched to testis by fibrous bands

Further exploration revealed a 3 × 3 cm globular paratesticular mass, attached to the testis by a fibrous band (Figure 2). The mass was meticulously dissected and completely separated from the testis. As the lesion was excised without injury to the tunica albuginea, an intraoperative decision was made to preserve the testis.

**Figure 3:**
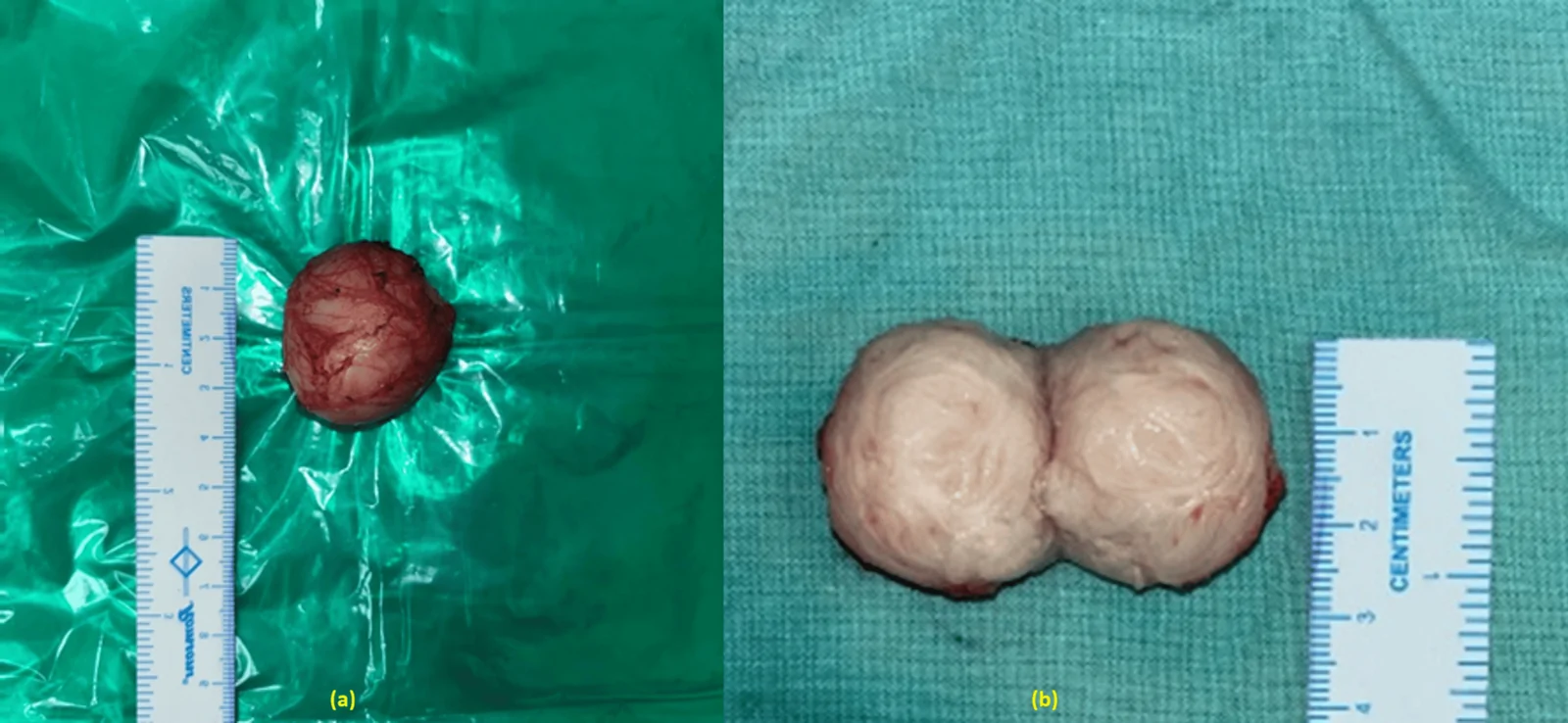
Gross specimen of excised left-sided paratesticular mass showing intact specimen (a) and cut section (b) with whorled pattern respectively.

Gross examination of the excised specimen revealed a firm, rubbery, whitish mass with a characteristic whorled appearance on cut section (Figure 3).

**Figure 4:**
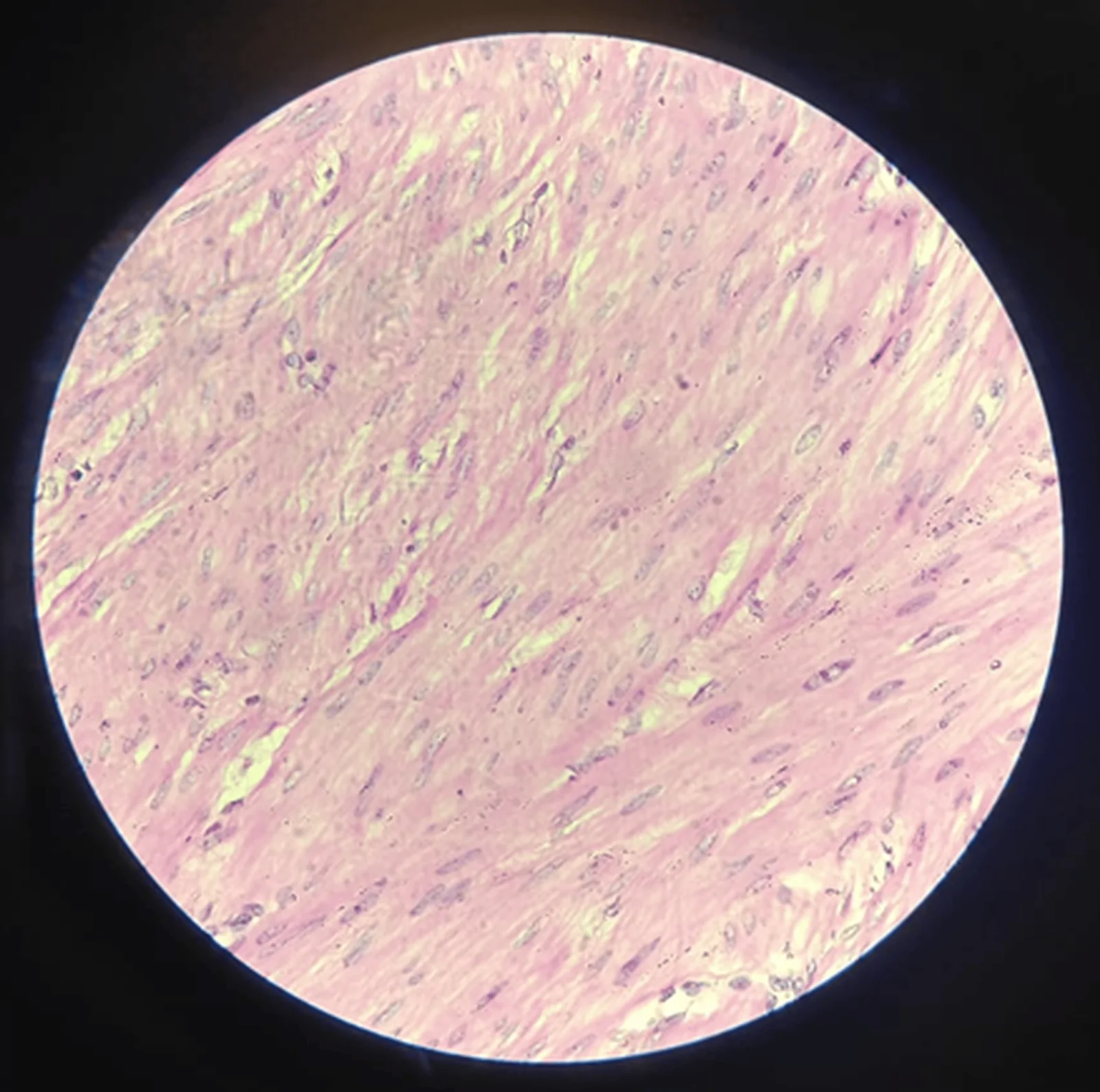
Microscopic examination showing uniform individual spindle-shaped tumor cells with cigar-shaped nuclei and moderate to abundant eosinophilic cytoplasm.

Microscopic examination showed a well-circumscribed tumor composed of interlacing fascicles and bundles. The tumor cells were uniform, spindle-shaped with blunt ends, exhibiting cigar-shaped nuclei and moderate to abundant eosinophilic cytoplasm (Figure 4). These histomorphological features were consistent with a benign spindle cell tumor, suggestive of leiomyoma.

The patient had an uneventful postoperative recovery. The surgical site healed well without any complications, and the patient remained asymptomatic on follow-up. No evidence of recurrence or residual lesion was noted during the early postoperative period.

## Discussion

Paratesticular tumors represent a small subset of intrascrotal masses, accounting for less than 5% of all intrascrotal lesions, with the majority originating from the spermatic cord, epididymis, or tunica vaginalis [1,4]. Although most paratesticular tumors are benign, their clinical presentation often mimics that of malignant testicular neoplasms, making accurate preoperative diagnosis challenging.

Leiomyoma is a benign smooth muscle tumor that rarely arises in the paratesticular region. When present, it is believed to originate from smooth muscle elements of the epididymis, spermatic cord, tunica albuginea, or tunica vaginalis [2]. Paratesticular leiomyomas typically occur in middle-aged to elderly men and commonly present as a slow-growing, painless scrotal mass, consistent with the presentation observed in the present case [3].

Ultrasonography remains the cornerstone of initial evaluation for scrotal masses. Paratesticular leiomyomas usually appear as well-circumscribed, solid, extratesticular lesions with variable echogenicity and mild internal vascularity on color Doppler imaging [5]. However, these imaging characteristics are nonspecific and may overlap with other benign entities such as adenomatoid tumors or lipomas, as well as malignant tumors including sarcomas. Cross-sectional imaging with CT or MRI can assist in confirming the extratesticular origin of the lesion and assessing for local invasion or distant spread, though definitive diagnosis relies on histopathological examination [6].

Serum tumor markers, including AFP, β-HCG, and LDH, are typically within normal limits in paratesticular leiomyomas. While normal markers help exclude germ cell tumors, they do not entirely rule out other malignancies, often necessitating surgical exploration [7].

The standard treatment for paratesticular leiomyoma is complete local excision. Testis-sparing surgery is strongly advocated when the tumor is well circumscribed, clearly separable from the testis, and intraoperative findings do not suggest malignancy [1]. This approach minimizes morbidity and preserves testicular function. However usually in testicular lumps an inguinal incision is preferred to avoid the risk of skin seeding, but in this case since the mass was separate from the testis as per clinical examination as well as radiological findings, the decision was made to take a scrotal incision. In the present case, meticulous dissection allowed complete excision of the tumor without injury to the tunica albuginea, enabling successful testicular preservation.

Grossly, leiomyomas characteristically exhibit a firm, rubbery consistency with a whorled cut surface. Histopathological examination typically reveals interlacing fascicles of uniform spindle cells with cigar-shaped nuclei and abundant eosinophilic cytoplasm, without features of malignancy such as mitotic activity, necrosis, or cellular atypia [8]. These features help distinguish leiomyoma from malignant spindle cell tumors, particularly leiomyosarcoma.

The prognosis following complete excision of paratesticular leiomyoma is excellent, with recurrence being exceedingly rare [9]. Awareness of this rare benign entity is therefore essential to prevent overtreatment and avoid unnecessary radical orchidectomy.

## Conclusions

Paratesticular leiomyoma is a rare benign tumor that should be considered in the differential diagnosis of painless extratesticular scrotal masses in middle-aged men. Accurate preoperative assessment using imaging and tumor markers, combined with careful intraoperative evaluation, allows for testis-sparing surgical excision. Histopathological examination remains the cornerstone for definitive diagnosis. Early recognition of this entity is crucial to prevent unnecessary radical surgery and ensure optimal patient outcomes.
